# RNA-Binding Proteins in Plant Immunity

**DOI:** 10.4061/2011/278697

**Published:** 2011-08-01

**Authors:** Virginia Woloshen, Shuai Huang, Xin Li

**Affiliations:** ^1^Michael Smith Laboratories, University of British Columbia, Vancouver, Canada V6T 1Z4; ^2^Department of Botany, University of British Columbia, Vancouver, Canada V6T 1Z4

## Abstract

Plant defence responses against pathogen infection are crucial to plant survival. The high degree of regulation of plant immunity occurs both transcriptionally and posttranscriptionally. Once transcribed, target gene RNA must be processed prior to translation. This includes polyadenylation, 5′capping, editing, splicing, and mRNA export. RNA-binding proteins (RBPs) have been implicated at each level of RNA processing. Previous research has primarily focused on structural RNA-binding proteins of yeast and mammals; however, more recent work has characterized a number of plant RBPs and revealed their roles in plant immune responses. This paper provides an update on the known functions of RBPs in plant immune response regulation. Future in-depth analysis of RBPs and other related players will unveil the sophisticated regulatory mechanisms of RNA processing during plant immune responses.

## 1. Introduction

**Figure 1:**
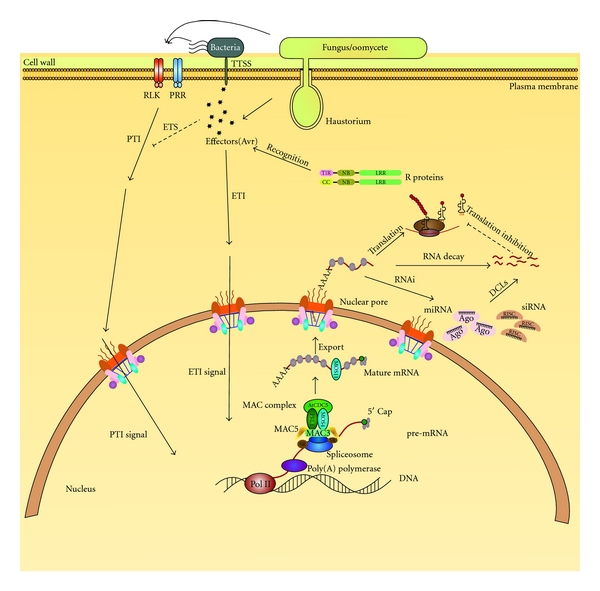
RNA processing steps that regulate plant immune responses. Pathogen associated molecular patterns (PAMPs) are recognized by pathogen recognition receptors (PRRs), which induce signaling cascades and lead to PAMP triggered immunity (PTI). To overcome this, bacterial, oomycete or fungal pathogens release effector molecules that inhibit PTI; this leads to effector-triggered susceptibility (ETS). Plants have evolved Resistance (R) proteins that recognize the effectors and lead to effector-triggered immunity (ETI). PTI and ETI both trigger downstream defence gene activation followed by RNA processing steps that include 3′ polyadenylation, splicing, 5′ capping and mRNA export. MAC5A/B associate with the MOS4-associated Complex (MAC), which associates with the spliceosome; therefore, MAC5A/B may contribute to mRNA splicing during pathogen defence [2]. MOS11 is also found in the nucleus; however, it is involved with mRNA export [3]. DCL2 and DCL4 are both required in the formation of siRNAs in RNAi, which assists in plant immunity [4, 5]. Similarly, AGO1, AGO2, and AGO7 are involved in RNAi, they are RNA-binding components of the RISC, which recruits the target mRNA [5, 6].

Plants have evolved complex pathogen defence mechanisms partly due to their sessile lifestyle and lack of mobile cells used by mammals. Each plant cell possesses an innate immunity system with which it can defend itself from pathogen attack [1]. Plant defence generally commences by the sensing of molecules or structural features possessed by the invading pathogen. These molecules, from bacteria, oomycetes, and fungi, often have conserved features termed pathogen-associated or microbial-associated molecular patterns (PAMPs or MAMPs). PAMPs are often recognized by transmembrane, pattern recognition receptors (PRRs) usually belonging to the receptor-like kinase (RLK) type, on the plant cell surface. Defence genes are then induced, initiating PAMP-triggered immunity (PTI), and pathogenesis is prevented. However, the pathogen may be able to surpass PTI, by releasing effector molecules, which would lead to effector-triggered susceptibility (ETS). Subsequently, the plants have evolved resistance (R) proteins that can recognize specific effectors and result in effector-triggered immunity (ETI). Recognition of effector molecules is accomplished by R proteins that most often contain nucleotide-binding (NB) and leucine-rich repeat (LRR) domains [1]. Once recognition occurs, a signalling cascade begins, leading to the activation of downstream genes to mount a robust and quick defence response to prevent the spread of pathogens (see Figure 1). 

Regulation of plant immunity is very complex. Multiple defence pathways exist downstream of R protein recognition. In general, upon recognition, effector signals are transmitted to the nucleus to promote defence gene expression. These target genes may code for transcription factors to trigger the transcription of downstream *Pathogenesis-Related* (*PR*) genes, enzymes needed for the synthesis of defence-related metabolites such as salicylic acid (SA). These signal transduction pathways typically leading to a hypersensitive response (HR) in which there is an accumulation of SA, reactive oxygen species, and the activation of *PR* genes that results in programmed cell death to halt pathogen invasion [7]. Although the aforementioned gene products have received most of the attention in research, there are many genes that have now been found to encode RNA-binding proteins (RBPs) that function at the posttranscription level [2–6, 8–14]. These RBPs provide hints to another level of regulation posttranscriptionally.

Gene regulation can occur at various points during the formation and delivery of the final mRNA product. Many genes are regulated at the level of transcription by either the activation or repression of gene transcription. However, in recent years it has become clear that regulation at the posttranscriptional level is just as prevalent. Posttranscriptional gene regulation allows for more rapid responses to environmental stimuli such as abiotic and biotic stresses in which a quick response would be beneficial or even crucial to the survival of the organism. Regulation at this level is partially made possible by RBPs, which facilitate RNA processing in various ways. 

Posttranscriptional RNA must be processed through several steps before it is ready for translation. Newly transcribed pre-mRNA must be 3′ polyadenylated, 5′ capped, edited, and spliced before it is considered mature mRNA to be exported to the cytoplasm and a candidate for translation (refer to Figure 1) [15]. Polyadenylation is among the first crucial steps in this process. Lack of polyadenylation prevents pre-mRNA from being spliced and subsequently translated [16]. Alternative polyadenylation has also been reported [17, 18]. 5′ capping and splicing must also occur prior to translation [15]. Splicing may also be alternative, allowing more information to be encoded by a single sequence of DNA [19]. The subsequent mature mRNA can then be differentially stabilized, exported, and delivered to ribosomes prior to translation [15, 20]. RBPs are essential for all steps of RNA processing. They are characterized by the presence of conserved RNA-binding motifs and are predicted to execute its function through binding with its RNA targets. Many RBPs have been characterised in various eukaryotes. However, in this mini-paper, we will focus on specific plant RBPs, that function in RNA processing and plant defence against microbial pathogens.

## 2. RBPs Involved in Plant Immunity

There have been a limited number of RBPs characterized in plants [15]. Even fewer have been assessed for their contribution to plant immunity. Several RBPs not only contain RNA binding domains and therefore have putative functions in RNA processing, they are also implicated in plant immune responses, as summarized in Table 1. Although much more research is required in this area, the following represents our current knowledge of RBPs related to plant pathogen defence against microbial pathogens, which is broadening our view of the importance of RNA processing in regulating plant defence responses.

### 2.1. PRP-BP

PvPRP1 is a proline-rich cell wall protein and is downregulated when exposed to a fungal elicitor [21]. To determine the cause of *PvPRP1* destabilization and subsequent degradation, proteins that specifically bind *PvPRP1* mRNA were isolated using RNA-protein UV cross-linking assays [8]. One 50 kD protein was found to bind *PvPRP1* mRNA, PRP-BP (PvPRP1-binding protein). PRP-BP binds *PvPRP1 *mRNA specifically in the 3′ region between nucleotides 855 and 1111. This potential binding region was further reduced to PRP940-967 using ^32^P-labelled, 5′ deletion clones. The sequence of PRP940-967 is rich in uracil (U) (refer to Table 1). Competition titrations were conducted using poly(U), poly(U-A), poly(A), poly(G), and poly(C); poly(U) was the only ribohomopolymer which is able to effectively compete with *PvPRP1* for PRP-BP binding. An RNA band shift analysis was also conducted to confirm PRP-BP binding at the U-rich region of *PvPRP1*.

The RNA-binding activity of PRP-BP was found to increase with acidity [8]. This is due to the reduction of sulfhydryl groups, which was determined by a binding assay using SH oxidizing and alkylating agents. Binding activity also increased following treatment with a fungal elicitor derived from *Colletotrichum lindemuthianum*.

These results implicate PRP-BP in plant defence. During pathogen infection, certain genes are expressed to assist in the protection of the plant such as cell wall strengthening genes, some of which encode proteins that are rich in both tyrosine and proline and strengthen the cell wall through isodityrosine cross-linking [38]. It is thought that the PvPRP1 protein is downregulated due to its low concentration of tyrosine and its lack of contribution to cell wall strengthening [21], which is accomplished by PRP-BP [8]. The binding activity of PRP-BP was found to be redox-regulated *in vitro*, in which the binding affinity changed depending on the redox state of sulfhydryl groups [8]. This is not surprising considering the redox changes that occur upon pathogen infection such as the production of H_2_O_2_ and SA [1]. This study illustrates a model where fungal elicitors can lead to the increased binding affinity of a RBP to its target mRNA, in this case by changing the redox state that allows such an interaction and subsequently degradation of the target mRNA. 

### 2.2. tcI14

One protein that has been implicated in RNA processing and plant defence is “tcI14” (tobacco cryptogein-induced) [9]. To identify mRNA transcripts that accumulate due to plant defence responses, tobacco plants were treated with a fungal elicitor found in *Phytophthora* species, cryptogein, which causes HR in tobacco [39]. 5′ rapid amplification of cDNA ends (5′ RACE) was used to isolate the full-length cDNA from genes that were activated following elicitor treatment. mRNA differential-display reverse-transcription PCR (DDRT-PCR) was then used to clone *tcI14*, which is induced by elicitor treatment. Sequence analysis revealed that the corresponding protein is homologous to human and *Drosophila* transformer-2-like SR-related (serine/arginine related) ribonucleoproteins. It contains two consensus sequences, RNP1 and RNP2, which are conserved RNA-binding domains [40]. tcI14 also contained an SR-rich repeat and an SR-rich region, commonly found in some splicing factors (refer to Table 1) [9]. 

The target RNA of “tcI14” is not yet known and confirmation of its RNA-binding activity has not been conducted. However, transformer-2-like ribonucleoproteins have been implicated in pre-mRNA splicing [41]. Due to the induction of “tcI14” upon cryptogein elicitation and its putative role as a transformer-2-like ribonucleoprotein, it likely contributes to utilizing splicing as a regulatory step in defence response against fungal infection.

### 2.3. GaPR-10

*GaPR-10* encodes a PR-10-like protein, which is a highly acidic protein with RNase activity [42]. *GaPR-10* was isolated from an expressed sequence tag (EST) cDNA library of cotton (*Gossypium arboretum*) that was exposed to *Verticillium dahliae*, a soil borne pathogenic fungus that causes wilt [10]. Sequence analysis revealed that the protein encoded by this gene was most likely cytoplasmic due to a lack of a signal peptide. It is homologous to other plant PR-10 proteins in *Betula*, *Phaseolus*, *Petroselinum*, *Sorghum*, and *Asparagus* genera. There is a C-terminal helix of the consensus sequence KAXEXYL and a P-loop structure of GDASPGSIVK that is suspected to bind RNA substrate (refer to Table 1).

To assess the RNase activity of GaPR-10, the protein was His-tagged at the N-terminus and expressed in *E. coli* [10]. RNase activity was tested using affinity-purified GaPR-10 incubated with yeast RNA. RNase activity was only observed after His-tag cleavage. Site-directed mutagenesis was also conducted, in which single amino acid replacements were made within the P-loop conserved region or residues suspected to be involved in the RNase catalytic reaction. All mutants were found to have decreased RNase activity. The results from this study indicate that the hydroxyl group of Tyr^150^ and the carboxyl group of Glu^148^ in the C-terminal helix are crucial for the RNase enzymatic activity, whereas the Gly^51^ and Lys^55^ residues of the P-loop are not essential.

RNA gel blot analysis of *GaPR-10* revealed that it is only expressed in roots of seedlings. However, after treatment with elicitors from *V. dahliae*, *GaPR-10* expression increased in roots and was also present in hypocotyls. Expression was also observed in suspension cultured cells after treatment with *V. dahliae*, as well as when treated with jasmonic acid (JA), a signalling molecule thought to be involved in plant necrotrophic pathogen resistance [10]. Expression of *GaPR-10* was found to be gradual after elicitation, which supports the hypothesis that *GaPR-10* functions to selectively degrade target RNAs that are produced during pathogen infection, which allows the host organism to return to a homeostatic state once the infection has ceased.

### 2.4. GRP7

*GRP7* (*Glycine-Rich Protein 7*) was isolated from an *Arabidopsis *cDNA library [22]. This protein contains RNP-CS-type RNA-binding domain, which is an RNA recognition motif (RRM) (refer to Table 1). Two types of *AtGRP7* transcripts are found in wild-type plants, the more abundant transcript contains both exons of the gene, whereas the less abundant transcript has both exons as well as the intron between them. Experiments were conducted to determine if GRP7 binds and regulates its own mRNA transcript [11]. GRP7 overexpression lines, *AtGRP7*-ox, were generated by overexpression with the 35S CaMV (cauliflower mosaic virus) promoter. GRP7 was found to bind its own mRNA at the 3′ UTR and the second half of its intron. Excess GRP7 activates an alternative 5′ splice site in which the second half of the intron is spliced out, resulting in the presence of a third transcript containing both exons separated by the first half of the intron. Downregulation of the regularly spliced transcript was also observed in overexpression plants. Replacement of regularly spliced transcript with the alternate transcript depends on high levels of functional GRP7 protein, since mutated overexpressed GRP7 protein did not activate the alternative splice site, and only the original two types of transcript were detected. Therefore, GRP7 is needed to regulate its own transcript. At high levels of GRP7 protein, the alternate transcript was produced but quickly degraded. It has a half-life of only thirty minutes in comparison to the four hours of regularly spliced transcript. Overexpressed GRP7 was also found to regulate mRNA of a related protein GRP8; however, it does not seem to regulate other glycine-rich proteins such as *AtU1*. GRP7 is hypothesized to accumulate during the circadian cycle; once levels reach a threshold, GRP7 binds its own mRNA and causes the production of an unstable alternative transcript resulting in a decrease of functional protein. These results implicate GRP7 in RNA processing, specifically stability control and possibly alternative splicing of its own mRNA [11].

GRP7 was found to be ADP-ribosylated by *hopU1*, a pathogen effector of *Pseudomonas syringae *pv. *tomato* DC3000 encoding an ADP-ribosyl transferase [43]. This ribosylation occurs at arginine residues within the RNP1 region of the RRM of the protein. This discovery implicates GRP7 in the plant immune response and how pathogen can target an RBP to achieve pathogenicity. *grp7* T-DNA insertion mutants of *Arabidopsis thaliana* were used to test for susceptibility to *P.s.t.* DC3000 and were shown to be more susceptible than wild type plants, confirming that GRP7 is required for pathogen defence. However, its exact role in RNA processing during plant defence awaits further research.

### 2.5. eIF4E

Eukaryotic initiation factor 4E (eIF4E) is highly conserved across eukaryotic kingdoms [15]. They associate with the 5′ cap-binding complex (CBC), which binds the 5′ mRNA immediately after its formation and contributes to splicing as well as mRNA export out of the nucleus. Cap binding by this complex is then transferred to eIF4E, which assists in the recruitment of other initiation factors that allow translation to occur by removing the secondary structure of the 5′ UTR [15]. eIF4E has been implicated as a susceptibility factor against viral pathogens [12, 23].

eIF4E binds the genome-linked proteins (VPg) of various potyviruses [23]. Potyviruses cannot autoreplicate and require the use of host proteins such as eIF4E for replication and therefore successful infection. One study examined the susceptibility of *Arabidopsis * mutants *Ateif(iso)4e*, mutated by transposon insertion, to *Turnip Mosaic Virus *(*TuMV*) as well as *Lettuce Mosaic Virus* (*LMV*)*, Tomato Black Ring Virus *(*TBRV*), and *Cucumber Mosaic Virus *strain *R *(*CMV-R*) [23]. *Ateif(iso)4e* mutant plants did not display viral symptoms in response to infection with *TuMV*, whereas wild-type plants are susceptible. This was confirmed using ELISA, RT-PCR, and backinoculation; *TuMV* was unable to replicate in *Ateif(iso)4e* mutants [23]. *Ateif(iso)4e* mutants were also challenged with *LMV*-Most, which infects wild-type *Arabidopsis *of the Columbia ecotype. Similar results were obtained as when inoculated with *TuMV*, therefore *AteIF(iso)4E* is required for replication of both potyviruses tested [23]. To determine if *AteIF(iso)4E *is required for replication of viruses other than potyviruses, *Ateif(iso)4e* was inoculated with *TBRV*, a nepovirus that also has VPg and *CMV-R*, a cucumovirus. Both viruses only mildly infect wild-type *Arabidopsis*; however, similar symptoms occurred in *Ateif(iso)4E* mutants; therefore, *AteIF(iso)4E* is not required for viral replication of other viruses such as *TBRV* and *CMV-R *[23]. AteIF(iso)4E seems to be specifically required for replication of certain potyviruses. 

Another study was conducted to determine if different forms of *Arabidopsis* eIF4E is required for potyvirus infection of *clover yellow vein virus* (*ClYVV*) and *turnip mosaic virus* (*TuMV*) [12]. Mutants of *AteIF4E* were generated by TILLINGs (targeting-induced local lesions IN genomes) and by obtaining T-DNA insertional mutants from the *Arabidopsis* stock centre (SALK-145583). Mutants were also generated for an isoform of this gene *AtIF(iso)4e* by transposon tagging and PCR screening. Using GFP-tagged *ClYVV* successful infection was detected in *Ateif(iso)4e *mutants, however, not in *Ateif4e* mutants. This indicates AteIF4E is needed for successful *ClYVV* viral infection. Susceptibility to another virus was also tested. when *TuMV* was used to infect both *Arabidopsis* mutants. Unlike *ClYVV*, *Ateif(iso)4e* mutants did not appear to be susceptible to *TuMV*, confirmed by RT-PCR. *Ateif4e* mutants were however susceptible to *TuMV*, which was determined by RT-PCR of RNA extracts of treated plants to detect *TuMV* RNAs. Therefore, different members of eIF4E seem to be selectively involved in infection by different potyviruses [12]. 

*eIF4E* genes form a gene family with redundant functions; thus one member may be rendered nonfunctional without detrimental effects on RNA processing or development [24]. Tomato plants have two homologs of *eIF4E*, *Sl-eIF4E1,* and *Sl-eIF4E2*, as well as the isoform *eIF(iso)4E*, each of which were mutated using TILLING [24]. All mutants were challenged with various potyviruses including two strains of *Potato Virus Y* (*PVY-LYE90* and *PVY-LYE84*), *Pepper Mottle Virus *strain *Texas *(*PepMoV-Texas*), and *Tobacco Etch Virus *(*TEV*). One mutant line of *Sl-eIF4E1* was found to be resistant to *PYV-LYE90* and *PepMoV-Texas* potyviruses due to a point mutation that ultimately prevents cap binding; however, these mutants were susceptible to *PYV-LYE84* and *TEV *[24]. These mutants were homozygous for the mutation and yet showed no deficiencies in development. Since *eIF4E* family members serve as susceptibility factors for potyviruses, mutating various forms of the RBP eIF4E could be a useful strategy to engineer potyvirus-resistant crops.

### 2.6. MOS2

*SNC1* carries a gain-of-function mutation in a TIR-NBS-LRR-type *R* gene. Constitutive activation of defence responses in *snc1* leads to autoimmune phenotypes such as dwarfism, high SA, heightened *PR* gene expression, and enhanced resistance against pathogens [44]. To search for components required for R protein-mediated immunity, a *modifier of snc1* (*MOS*) genetic screen was conducted. *mos2-1* mutant was identified from the screen using a *snc1 npr1* double mutant background with fast-neutron mutagenesis. It no longer exhibited the *snc1*-related phenotypes [13]. A map-based cloning approach was employed to isolate the *MOS2* gene. MOS2 contains one G-patch domain at the centre and two KOW motifs near the C-terminus as revealed by sequence analysis (refer to Table 1). MOS2 is evolutionarily conserved among multicellular organisms, with homologs in human, mouse, and *C. elegans* [13]. G-patch domains have been found in RNA-binding proteins in other organisms and are thought to contribute to RNA-protein interactions [25]. KOW motifs are capable of binding both RNA and proteins using different residues within the domain [26].

Upon infection with virulent bacteria *Pseudomonas syringae *pv. *maculicola* (*P.s.m.*). ES4326 or oomycete *Hyaloperonospora arabidopsidis* (*H.a.*) Noco2, *mos2 snc1 npr1 *mutants displayed increased susceptibility compared to *snc1 npr1* mutants [13]. SA level was also partially suppressed in the triple mutant. To determine if *MOS2* is required for basal resistance, single mutant *mos2* plants were inoculated with *P.s.m. *ES4326. Mutant plants were more susceptible to this pathogen. To determine if *MOS2* is required for *R* gene-mediated resistance single mutants were inoculated with *P.s.m.* ES4326 *AvrB* and *P.s.t.* DC3000* AvrRPS4,* corresponding to different *R* genes in *Arabidopsis*. Mutants were also susceptible to these pathogens. Therefore, *MOS2* is required for both basal resistance and *R* gene-mediated resistance [13].

The contribution of *MOS2* to RNA processing and pathogen resistance has not been fully characterized, nor has its homologs found in other organisms. However, based on its RNA-binding domains and its increased susceptibility to pathogens when mutated, *MOS2* is suspected to play a crucial role in RNA processing and plant pathogen defence. GFP-fusion analysis shows MOS2 localizes to the nucleus indicating that it probably contributes to RNA processing prior to mRNA export; however, its exact role in RNA processing has yet to be clarified [13].

### 2.7. MOS11

As with *MOS2*, *MOS11 *was found during the *MOS *screen previously mentioned [13]. *mos11-1* was identified in a T-DNA-mutagenized *snc1* population, and when mutated, suppressed the *snc1* phenotypes [3]. Sequence analysis revealed that MOS11 is similar to human CIP29, a cytokinin-induced protein that has been shown to bind RNA and localize to the nucleus (refer to Figure 1 and Table 1) [27]. 

Semiquantitative RT-PCR was performed on *mos11 snc1 *mutants, which revealed that *PR1* and *PR2* expression was greatly reduced in comparison to *snc1* [3]. SA levels were also significantly decreased. Susceptibility of *mos11 snc1* mutants was tested through infection with the oomycete pathogen *H. a. *Noco 2 and the bacteria *P.s.m. *ES4326. There was intermediate susceptibility to the oomycete pathogen and the bacterial pathogen; therefore, *mos11* only partially suppresses the *snc1* phenotypes. GFP-fusion analysis showed that MOS11 localized to the nucleus. MOS11 is homologous to human CIP29, which has RNA-binding activity [27]. CIP29 interacts with RNA helicases DDX39 and FUS/TLS. DDX39 is homologous to a *Drosophila *RNA helicase which is involved in RNA export [45]. Due to this homology, the role of MOS11 in mRNA export was tested by poly(A) *in situ* hybridization [3]. There was higher accumulation of mRNA in the nucleus of *mos11* plants compared with wild type; however, it does not appear that mRNA export was completely compromised [3].

The homology of MOS11 with human CIP29 implicates MOS11 in RNA binding as well as protein binding, potentially forming complexes with other proteins. The MOS11 protein sequence does not possess any known RNA-binding domains; however, the highly conserved positively charged amino acids in MOS11 could potentially bind mRNA. MOS11's exact role in RNA export awaits further investigation. 

### 2.8. DCL2 and DCL4

Dicer proteins are highly conserved across kingdoms and involved in RNA interference (RNAi), a posttranscriptional gene silencing mechanism (refer to Figure 1) [29, 46]. Four Dicer-like (DCL) proteins have been characterized in *Arabidopsis* [47]. DCL1, DCL2, DCL3, and DCL4 all contain a helicase, PAZ, RNase III-like, and double-stranded RNA-binding domains (refer to Table 1) [28]. DCL1 functions in regulating developmental genes [47]. DCL3 functions in heterochromatin structure regulation [4, 29]. DCL2 is required for siRNA formation for viral RNA silencing [4], and DCL4 is required for sense transgene-induced RNA-silencing and production of siRNAs for endogenous gene regulation [28]. Only DCL2 and DCL4 are suspected to contribute to plant defence against both viruses and fungi [4, 5]. 

*Arabidopsis* T-DNA insertional mutants *dcl2* and *dcl4* challenged with a viral pathogen, *Tobacco rattle virus* (TRV-PDS), to which wild-type *Arabidopsis* plants are immune. *dcl2* mutants showed no change in siRNA accumulation, whereas in *dcl4* mutants, 22-nucleotide siRNAs replaced the typical 21-nucleotide siRNAs found during viral pathogen infection [4]. No viral RNA accumulated in single mutants indicating they may be functionally redundant. To further test this, double mutants were created by crossing the two single mutants. The double mutant *dcl2 dcl4* accumulated 24-nt siRNAs, had viral RNA accumulation, and exhibited viral disease symptoms. Therefore, the 22-nt and 21-nt siRNA produced by DCL2 and DCL4, respectively, contribute to silencing TRV RNA [4]. 

Another study used the same mutants, *dcl2 *and *dcl4* to test resistance against the soil born fungi *Verticillium dahliae* [5]. *dcl2* mutants had no significant difference in susceptibility in comparison to wild-type plants, whereas *dcl4* mutants showed enhanced susceptibility in terms of disease symptoms such as necrosis and fungal biomass determined by qRT-PCR [5]. 

These two studies indicate that the RBP DCL4 contributes to plant defence against both viral and fungal pathogens due to the increase in susceptibility when DCL4 is rendered nonfunctional. Whereas DCL2 has only been implicated in defence against viral pathogens as there was no difference in susceptibility to the fungus *V. dahliae* [4, 5]. This illustrates the importance of RBPs in plant defence through RNAi-mediated mechanisms.

### 2.9. AGO1, AGO2, and AGO7

 Argonaute (AGO) proteins are also involved in RNAi [29, 46]. Specifically, AGO proteins are RBPs in the RNA-induced silencing complex (RISC) of RNAi (refer to Figure 1) [29]. AGO proteins directly bind RNA, which allows RISC to either cleave the target RNA to be silenced or prevents translation [29]. All AGO proteins have a PAZ domain, which binds the 3′ two nucleotide overhang that is created earlier in the RNAi pathway. PIWI is another conserved domain, which has nuclease activity (refer to Table 1) [30].

RNAi is known to function in plant defence in response to both viruses and recently bacteria; however, only AGO1, AGO2, and AGO7, have been shown to contribute to plant defence [5, 6, 31, 32]. To determine if RNAi contributes to plant defence against fungi, mutants of RBPs required for RNAi, such as AGO1 and AGO7 were challenged with previously mentioned *V. dahliae,* the wilt-causing fungus [5]. *ago1* mutants were obtained using TILLING [33], and *ago7* mutants were T-DNA mutants obtained from the *Arabidopsis* stock centre [5]. Using fungal-specific primers in real-time quantitative PCR, the fungal biomass accumulation was assessed. *ago7 * mutants were more susceptible to *V. dahliae* and had greater stunting and necrosis in comparison to wild-type control plants. Fungal biomass was also much higher. However, *ago1* mutants were more resistant and displayed less necrosis and no anthocyanin production, biomass accumulation was also less than that found in wild-type plants. These indicate that different AGO proteins may have different roles in plant immunity.

More recently, expression analysis was conducted using RT-PCR on the *AGO* genes in response to *Pseudomonas syringae *pv. *tomato* (*P.s.t.*) DC3000 and *P.s.t. *(*avrRpt2*) [6]. Only *AGO2* transcripts increased; AGO2 protein level was assessed using western blot analysis to confirm this increase. To assess the role of AGO2 in defence responses, a bacterial infection assay was conducted on *ago2* mutants. Mutants were more susceptible to* P.s.t.* (*avrRpt2*), a pathogen with an avirulence factor corresponding to the *R* gene *RPS2*, implicating AGO2 in *R* gene-mediated defence. Mutants were also susceptible to *P.s.t.* (EV) in comparison to wild-type plants implicating AGO2 in basal defence. 

Partial redundancy was suspected with AGO2, AGO3, and AGO7 therefore, double and triple mutants were created by crossing single and double mutants. Double and triple mutants *ago2 ago7* and *ago2 ago3 ago7* were more susceptible to *P.s.t. *(*avrRpt2*) than single *ago2* or *ago7* mutants. *ago2 ago3* double mutants were similarly susceptible to the pathogen as *ago2* mutants, confirming AGO3 does not contribute to pathogen defence. The level of bacterial growth of *P.s.t. *(EV) was comparable in single, double, and triple mutants of *ago2*; AGO2 seems to be the only AGO involved in basal defence. *ago2* mutants were also susceptible to *P.s.t. *(*avrRpt2*), which triggers an *R* gene-mediated defence response. AGO2 is thus required for both basal and *R* gene-mediated defence [6].

Since AGO2 contributes to RNAi through the production of small single-stranded RNA molecules [35], a search for AGO2-associated sRNAs was conducted. Illumina deep sequencing was carried out following treatment with *P.s.t. *(*avrRpt2*). MicroRNA (miRNA) was thought to be nonfunctional as it forms miRNA::miRNA duplexes that are subsequently degraded by Dicer-like proteins and therefore cannot silence its target [35]. However, the most abundant sRNA associated with AGO2 postinfection was the miRNA, miR393b indicating miRNA may have biological function [6]. AGO1-associated miRNA was also monitored using immunoprecipitation. miR393b was only found in large quantities in the AGO2 fraction; however, its miRNA counterpart, miR393, which has been implicated in plant defence [34] was only found in the AGO1 fraction. Single mutants *ago2* had virtually no miR393b expression.

To elucidate the role of miR393b in plant immunity, possible target genes were predicted using the miR393b sequence. Of three possible targets, only *At5g50440*, which encodes MEMB12, a SNARE (soluble N-ethylmaleimidesensitive factor attachment protein receptor) protein involved in cytoplasmic trafficking and localized in the golgi, was tested. Coexpression analysis was conducted to confirm that MEMB12 is the target of miR393b; MEMB12 was down regulated in the presence of miR393b. MEMB12 proteins were much less abundant than its corresponding transcript; therefore, miR393b most likely downregulates MEMB12 by translational inhibition [6]. 

To determine the role of MEMB12 in plant immunity, *memb12* mutants were obtained with transposon tagging. Mutants were less susceptible to *P.s.t. *(*avrRpt2*) and *P.s.t. *(EV). MEMB12 localization assays were conducted, and it was found to localize to the Golgi. *memb12* mutants were also assessed for antimicrobial protein production, specifically PR1, a highly expressed protein during plant defence [48]. Intracellular PR1 was significantly higher in *memb12* plants than in wild-type plants following pathogen infection with *P.s.t. *(*avrRpt2*). *PR1* levels were low in *ago2* mutants. Other SNARE proteins were also tested such as SYP61 and SYP121; however, only MEMB12 was found to be responsible for vesicle trafficking of PR1. Therefore, AGO2 is implicated in plant immunity by binding to miR393b that targets *MEMB12*, which modulates PR1 exocytosis. To further confirm this link, miR393b overexpression lines were created and found to have a similar phenotype as *memb12* mutants such as enhanced resistance to *P.s.t. *(*avrRpt2*) and higher levels of *PR1* [6].

Several AGO proteins have now been implicated in plant defence [5, 6]. Mutations in *AGO1* and *AGO7*, essential RBPs of RNAi, showed differing levels of susceptibility to *V. dahliae* [5]. AGO2 was found to produce an miRNA responsible for downregulating a SNARE protein leading to increased PR1 secretion and enhanced resistance against virulent bacterial pathogens [6]. This implicates RNAi in plant defence not only against viral pathogens but also bacterial and fungal pathogens and illustrates the contribution of AGO1, AGO2, and AGO7 RBPs in this process. 

### 2.10. MAC5A/5B

MAC5A and MAC5B are two partially redundant RBPs that associate with the MOS4-associated complex (MAC) (refer to Figure 1) [2]. The MAC consists of many subunits including core MOS4, AtCDC5, MAC3A/3B, and PRL1 proteins that are highly conserved across kingdoms and form a nuclear complex that is important in plant immunity [36]. The MAC has also been implicated in splicing of mRNA as the homologous complexes in yeast and human have been shown to associate with the spliceosome, a complex involved in RNA splicing [49]. An attempt to identify other components that associate with this complex was conducted using complementing *MOS4-HA* transgenic lines in the *mos4* mutant background. Immunoaffinity purification using anti-HA microbeads followed by mass spectrometry proteomics analysis revealed that the MAC contains many protein constituents. MAC5A was further investigated with reverse genetics analyses. Sequence analysis revealed that MAC5A and MAC5B are highly homologous to each other and have a CCCH-type zinc finger as well as an RNA recognition motif (RRM) (refer to Table 1) [2]. *MAC5A* is expressed at a higher level than *MAC5B*.

Reverse genetics analyses were carried out using T-DNA insertional mutants of *mac5a* and *mac5b *[2]. MAC5A and MAC5B are partially redundant since only *mac5a* exhibited minor growth defects; however, double mutant is lethal. The *snc1* mutant background, which displays constitutive defence responses and increased resistance against pathogens [44], was used to assess the roles of these proteins in plant immunity. *snc1 mac5a* double mutant was more susceptible to infection than *snc1* by the oomycete pathogen *H. a. *Noco2 and the bacterial pathogen *P.s.t.* strain DC3000. *snc1 mac5b* mutants displayed a similar resistance to these pathogens as *snc1* single mutants. This lack of susceptibility is thought to be due to the inability of *mac5b* to suppress the dominant *snc1* phenotype, whereas *mac5a* suppresses *snc1* resistance further supporting the unequal redundancy of these two genes. Single mutants, *mac5a* and *mac5b,* did not exhibit enhanced susceptibility to the aforementioned pathogens nor were they susceptible to *R* gene-specific pathogens, *P.s.t. avrRps4* and *P.s.t. avrPphB*, which are typically recognized by the R proteins RPS4 and RSP5, respectively. This indicates that MAC5A/5B are not required for basal defence or R-protein-mediated defence other than *snc1* mediated immunity; however, this may be due to the redundancy of MAC5A and MAC5B, which cannot be easily teased apart because of double mutant lethality [2]. 

The roles of MAC5A/B and of the MAC in general in alternative splicing have yet to be demonstrated. However, its established role in plant immunity [36] and its association with known spliceosome components [2] implicate the MAC in the signal transduction pathway from R protein effector recognition ultimately leading to defence activation, possibly with assistance from the spliceosome. Therefore, MAC5A/B may be facilitating splicing to regulate their target defence gene mRNA.

### 2.11. AtRBP-DR1

RPS2 (resistance to *Pseudomonas syringae* 2) is a CC-type NBS-LRR R protein. Coimmunoprecipitation was used to identify proteins that associated with RPS2 in *Arabidopsis thaliana*, one of which was AtRBP-DR1 [37]. Mass spectrometry shows that AtRBP-DR1 has three RRMs, two at the N-terminus and one at the C-terminus (refer to Table 1). A T-DNA insertion mutant had no accumulation of *AtRBP-DR1 *mRNA [14]. It was used to test the role of AtRBP-DR1 in plant defence through infection with *P.s.t.* DC3000 to assess its role in basal defence, as well as strains with *AvrRpm1* or *AvrRpt2* to assess *R* gene-mediated resistance. Resistance was compromised in mutants infected with *P.s.t. *DC3000 with no Avr proteins; however, resistance remained when mutants were infected with bacteria containing Avr proteins. Therefore, AtRBP-DR1 contributes to basal resistance, however, does not contribute to *R* gene-mediated defence. This was confirmed with complementation analysis. Over-expression analysis was also conducted by transforming *Atrbp-dr1* mutants with *AtRBP-DR1* under the control of the 35S CaMV (cauliflower mosaic virus) promoter. These transformants had a dwarf phenotype that correlated with an increase in protein level as revealed by immunoblot analysis [14]. Transformants also had restored resistance against *P.s.t. *DC3000. The dwarf phenotype of mutants overexpressing *AtRBP-DR1* was probably due to an increase in SA production, which was determined by (q)RT-PCR using two genes involved in the SA pathway, *SID2 *(*SA induction deficient*) and *PR1*. The dwarf phenotype of overexpression lines was found to be *SID2 *dependant since *AtRBP-DR1*-ox *sid2* mutants were no longer dwarf and had little accumulation of *PR1* [14]. Taken together, AtRBP-DR1 seems to play a key role in regulating SA-mediated defence responses.

To further elucidate the function of the AtRBP-DR1 protein, YFP::HA-fusion analysis was carried out. AtRBP-DR1 seems to be localized in the cytoplasm; however, localization in the nucleus is also possible [14]. This localization data suggest that AtRBP-DR1 may play a role in transcript regulation postmRNA export. Despite the initial *in vitro* association between RPS2 and AtRBP-DR1 [37], this was not detectable *in vivo*; therefore, AtRBP-DR1 may not form a complex with RPS2 as previously hypothesized [14]. AtRBP-DR1 is suspected to function in RNA processing due to the presence of RRMs and is implicated in plant resistance against *P.s.t. *DC3000; however, its mRNA targets are not yet known and require further research. 

## 3. Conclusion

Regulation of plant defence at the level of RNA processing has emerged as an important aspect of plant immunity. RBPs have been implicated in almost every step of RNA processing during plant defence. Therefore, not only is plant defence regulated at the level of transcription, it is also fine-tuned at the level of post-transcriptional RNA processing. These examples represent only a small number of the putative RBPs that have been characterized. Genomic analysis has identified over 200 RBPs in the model plant *Arabidopsis thaliana*, fifty percent of which are unique to plants, most with unknown functions. The RBPs are orthologous to metazoan RBPs; however, the putative functions of most of these have not been studied [50]. Studying related RBPs of plants in response to pathogen infection may contribute to our understanding of similar RBPs in other kingdoms. RBPs are traditionally difficult to study due to the instability of their mRNA targets. The advent of next generation sequencing approaches such as RNA-seq will vastly enhance our capability to analyse RBPs and knockout mutants of RBPs.

## Figures and Tables

**Table 1:** Summary of RBPs in plant immunity.

RNA binding-Protein	Conserved RNA-binding motif	Other properties	Possible functions	Plant origin	References
PRP-BP		Binds U-rich region	PvPRP1 mRNA binding protein, may function in the elicitor-induced destabilization of *PvPRP7 *mRNA	Bean	[8]
tcI14	SR-rich repeat region RNP1 and RNP2		Alternative splicing	Tobacco	[9]
GaPR10		K-A-X-E-X-Y-L domainand a P-loop	Ribonuclease activity	Cotton	[10]
GRP7	RRM, glycine-rich motif RNP-binding domain		mRNA stability and control,pathogen defence	*Arabidopsis*	[11, 22]
eIF4E		Eukaryotic initiation factor 4E homolog domain	Components of 5′ cap binding complex (CBC), response to virus, translational initiation	*Arabidopsis*	[12, 23, 24]
MOS2	G-patch and KOW motif		RNA binding	*Arabidopsis*	[13, 25, 26]
MOS11		Homologus to human RNA binding protein CIP29	mRNA export	*Arabidopsis*	[3, 27]
DCL2	Double-stranded RNAbinding motif, Double stranded RNA binding domain	Helicase superfamily c-terminal domain, RIBOc. Ribonuclease III C terminal domain, PAZ domain	Defense response to virus, maintenance of DNA methylation, producing of ta-siRNAs involved in RNA interference	*Arabidopsis*	[4, 5, 28]
DCL4	Double-stranded RNA binding motif, Double stranded RNAbinding domain	Helicase superfamily c-terminal domain, RIBOc. Ribonuclease III C terminal domain, PAZ domain	RNA processing, defense response to virus, maintenance of DNA methylation, production of lsiRNA, siRNA and ta-siRNAs involved in RNAinterference,virus induced gene silencing	*Arabidopsis*	[4, 5, 28]
AGO1	PIWI domain, PAZ domain	DUF1785 domain, PLN03202 domain, Glycine-rich region of argonaut	RNA interference,gene silencing by miRNA, innate immune response, leaf morphogenesis, virus induced gene silencing	*Arabidopsis*	[5, 6, 29–34]
AGO2	PIWI domain, PAZ domain	DUF1785 domain, PLN03202 domain	siRNA binding, defense response to virus, RISC compoments	*Arabidopsis*	[5, 6, 29–32, 35]
AGO7	PIWI domain, PAZ domain	DUF1785 domain, PLN03202 domain	Nucleic binding, RISC components, gene silencing by miRNA, production of lsiRNA and ta-siRNAs involved in RNA interference	*Arabidopsis*	[5, 6, 29–32]
MAC5A/5B	CCCH-type zinc-finger domain, RNA recognition motif		Component of MAC, associated with spliceosome, defense response to bacterium	*Arabidopsis*	[2, 36]
AtRBP-DR1	Three RNA recognition motifs		Positive regulation of salicylic acid mediated signaling pathway	*Arabidopsis*	[14, 37]
